# Multicontamination Toxicity Evaluation in the Model Plant *Lactuca sativa* L.

**DOI:** 10.3390/plants13101356

**Published:** 2024-05-14

**Authors:** Veronika Zemanová, Marie Lhotská, Milan Novák, František Hnilička, Marek Popov, Daniela Pavlíková

**Affiliations:** 1 Department of Agroenvironmental Chemistry and Plant Nutrition, Faculty of Agrobiology, Food and Natural Resources, Czech University of Life Sciences Prague, Kamýcká 129, 165 00 Prague, Czech Republic; 2 Department of Botany and Plant Physiology, Faculty of Agrobiology, Food and Natural Resources, Czech University of Life Sciences Prague, Kamýcká 129, 165 00 Prague, Czech Republic

**Keywords:** anthropogenic contamination, bioaccumulation, metals/metalloids, nitrogen metabolism, oxidative stress, stress response

## Abstract

Many contaminated soils contain several toxic elements (TEs) in elevated contents, and plant–TE interactions can differ from single TE contamination. Therefore, this study investigated the impact of combined contamination (As, Cd, Pb, Zn) on the physiological and metabolic processes of lettuce. After 45 days of exposure, TE excess in soil resulted in the inhibition of root and leaf biomass by 40 and 48%, respectively. Oxidative stress by TE accumulation was indicated by markers—malondialdehyde and 5-methylcytosine—and visible symptoms of toxicity (leaf chlorosis, root browning) and morpho-anatomical changes, which were related to the change in water regime (water potential decrease). An analysis of free amino acids (AAs) indicated that TEs disturbed N and C metabolism, especially in leaves, increasing the total content of free AAs and their families. Stress-induced senescence by TEs suggested changes in gas exchange parameters (increase in transpiration rate, stomatal conductance, and intercellular CO_2_ concentration), photosynthetic pigments (decrease in chlorophylls and carotenoids), a decrease in water use efficiency, and the maximum quantum yield of photosystem II. These results confirmed that the toxicity of combined contamination significantly affected the processes of lettuce by damaging the antioxidant system and expressing higher leaf sensitivity to TE multicontamination.

## 1. Introduction

The origin of elevated concentrations of toxic elements (TEs) in soils is often associated with human-induced activity. Floodplain or riverine soils are prone to TE pollution resulting from anthropogenic activities (such as mining and ore smelting) and flooding events. Floodplain soils of the Litavka River (Příbram region, Czech Republic) are heavily multicontaminated by historic lead–silver mining and smelting activities and extreme flooding events that occurred at various time between 1932 and 2002 [1]. Analyses from this region have shown that the highest enrichment levels of soils are observed for Pb, Zn, Cd, and As [2,3]. The effect of soil multicontamination on plants is influenced by many factors, such as availability, mobility, and oxidation state of TEs in soil; interactions between elements; soil properties (pH, redox potential, texture, clay content, and soil organic matter); plant species and cultivars; and plant growth period [4,5,6]. Plants growing in TE-contaminated soils show visible symptoms of TE influence, such as inhibition of biomass accumulation, changes in leaf thickness, leaf chlorosis, senescence due to TE uptake, and morphological changes [7]. According to Ivanov and Zhukovskaya [8], TEs causing a slowdown of growth can be manifested in root development at the subcellular level (cell division, chromosomal aberrations, and mitotic anomalies (chromosomal abnormalities)). The influence of the presence of TEs can be observed in the anatomy of the roots. The root epidermis, cortical cells, and xylem vessels were negatively influenced by TEs in the study by Nazir et al. [9]. The overall deformation of root cells was also reported by Dey and Mondal [10]. According to DalCorso et al. [11], in plants, TEs caused the following four main processes: uptake competition between similar cations, direct interaction with the sulfhydryl group (-SH) of functional proteins, displacement of essential cations from specific binding sites leading to loss of function, and generation of reactive oxygen species (ROS). Plants initiate cellular defense mechanisms to reduce the adverse effects of TEs. The biosynthesis of molecules such as metallochaperones, organic acids, glutathione, phytochelatins, and flavonoid and phenolic compounds is among these mechanisms [12]. The disruption in the equilibrium of the redox system in cells induces increased ROS formation [13].

Bioaccumulation of TEs in the edible parts of vegetables cultivated in contaminated soils results in their entry into the food chain and represents a potential health risk [3]. TE uptake by vegetables can be classified as follows according to the TE concentrations in their edible parts: leafy/stem vegetables > root vegetables > tubers > fruiting vegetables/fruits/berries. Lettuce accumulates high TE concentrations in its leaves [14]. Mustățea et al. [15] confirmed the highest accumulation of metals (Cd, Cr, Pb, As) in lettuce compared with cabbage and green onions. For this reason, lettuce was chosen in our experiment as a model plant for observing the influence of TE on plant metabolism. The accumulation of TEs in plant biomass impairs various biochemical, physiological, and morphological functions, leading to stress-induced senescence in plants. Most published studies describe the effect of only one or two elements on plants, and a limited number focus on the influence of soil multicontamination. Therefore, this study evaluated the toxic effects of multicontaminated soil (As, Cd, Pb, and Zn) on plant metabolism. We identified the main changes in the physio-biochemical attributes affecting the metabolism and growth of lettuce. As a result of multicontamination, we assumed the changes in ROS stress markers, specifically malondialdehyde (MDA) and DNA methylation expressed by the content of 5-methylcytosine (5-mC), and changes in free amino acid content and photosynthetic parameters. It was also possible to assume a negative influence of multicontamination on root morphology and anatomy.

## 2. Results

### 2.1. Accumulation of Toxic Elements in Lettuce

Among the monitored TEs (As, Cd, Pb, and Zn), only Cd and Zn were detectable in lettuce leaves and roots of the control group (Table 1). The accumulation of both elements in the leaves and roots of lettuce in relation to the content of Cd and Zn in the soil was evaluated using the bioconcentration factor (BCF). The BCF values for Cd were >1, while these for Zn were <1 (Table 1). The Cd content reached similar values in both organs of the lettuce, while the Zn content was approximately 2.5-fold higher in the roots than in the leaves. This trend for Cd and Zn confirmed the translocation factor (TF), which reached a value of 0.96 for Cd and 0.40 for Zn. In the control, other toxic elements—As and Pb—were below the limits of detection, making it impossible to calculate the TF and BCF.

The data in Table 1 showed that all monitored TEs of the multicontamination group were above the detection limit and accumulated more in the lettuce roots compared with the leaves. This was confirmed by the TF for As, Cd, Pb, and Zn, which reached values of 0.93, 0.70, 0.64, and 0.97, respectively. A significant difference was confirmed for Cd and Pb. Compared with the leaves, the Cd and Pb content in the roots was approximately 1.4- and 1.6-fold higher, respectively. However, the As and Zn content reached a similar range of values in the roots and leaves of the multicontamination group (Table 1).

### 2.2. Biomass Production, Morphology, and Root Anatomy of Lettuce

**Figure 1:**
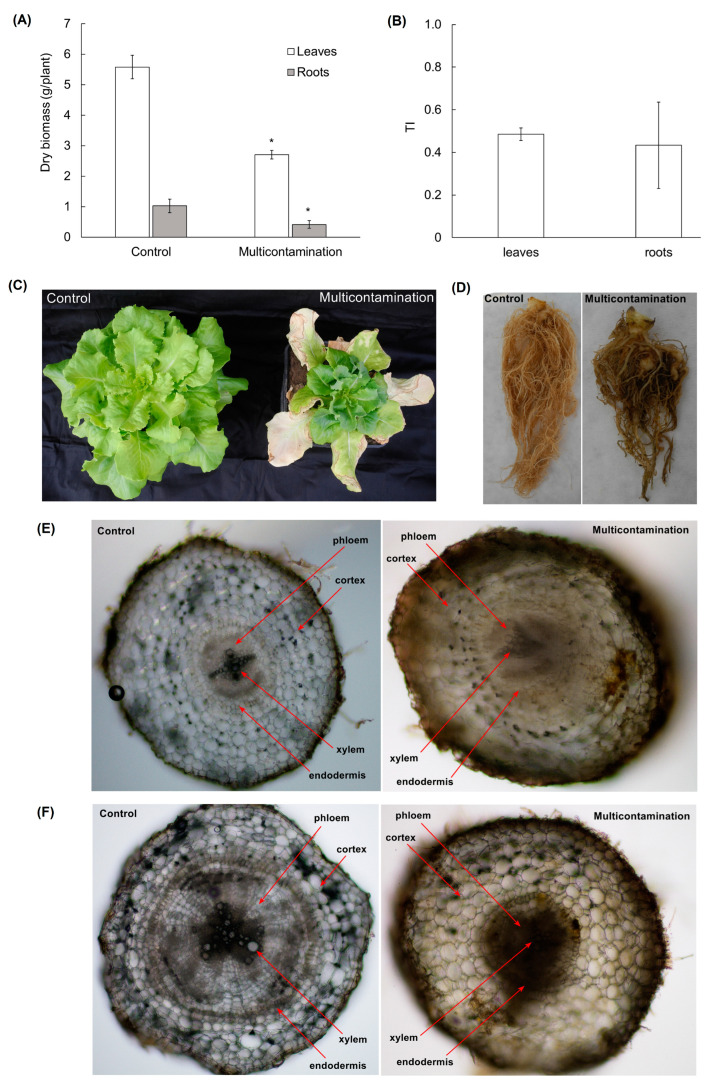
Dry biomass of lettuce from the control and multicontaminated soil (**A**). Values represent means ± SD of four biological replicates. Asterisks indicate significant differences (*p* ≤ 0.05) between treatments (control × multicontamination) for lettuce organs based on Fisher’s LSD test. Tolerance index (TI, expressed as the ratio of multicontamination dry biomass to control dry biomass) for lettuce leaves and roots (**B**). Values represent means ± SD of four biological replicates without significant difference between organs (leaves × roots). Morphology of lettuce from the control and multicontaminated soil (**C**,**D**). Cross-section through the tap roots (**E**, 100× magnification) and lateral roots (**F**, 100× magnification) of lettuce from the control and multicontaminated soil.

The accumulation of As, Cd, Pb, and Zn in the multicontamination lettuce significantly decreased the growth of leaves and roots (Figure 1A). Compared with the control, the production of leaf and root dry biomass in the multicontamination group was 2.5- and 2.0-fold lower, respectively. The negative effect of multicontamination on the biomass of lettuce confirmed the tolerance index (TI), which reached a value of 0.49 for leaves and 0.43 for roots (Figure 1B). Both organs were similarly affected, with no statistically significant difference between leaves and roots. Furthermore, leaves and roots of the multicontamination group showed changes in morphology and visual damage, including chlorosis, browning, and necrosis (Figure 1C,D).

To identify changes in the anatomy of the roots, a cross-section analysis of the lettuce tap and lateral roots was performed (Figure 1E,F). Both the tap and lateral roots of the multicontamination group showed different coloration compared with the control group. Changes in color might have been related to the lignification of root tissues and/or a symptom of incipient necrosis. Additionally, a difference was observed in the differentiation of the secondary xylem and in the development of the endodermis, especially the Casparian strips.

### 2.3. Malondialdehyde and 5-Methylcytosine Content in Lettuce

**Figure 2:**
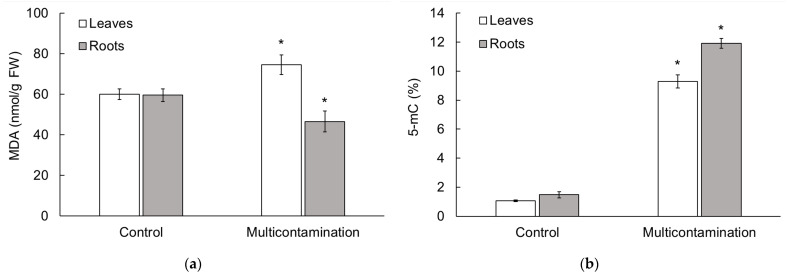
(**a**) Malondialdehyde content (nmol/g FW) in lettuce roots and leaves; (**b**) 5-methylcytosine content (% FW) in lettuce roots and leaves from the control and multicontaminated soil. Values represent means ± SD of four biological replicates. Asterisks indicate significant differences (*p* ≤ 0.05) between treatments (control × multicontamination) for lettuce organs based on Fisher’s LSD test. MDA—malondialdehyde, FW—fresh weight, 5-mC—5-methylcytosine.

To identify the stress response to As, Cd, Pb, and Zn accumulation, we determined the content of MDA (Figure 2a) and 5-methylcytosine (5-mC, Figure 2b) in lettuce leaves and roots. The MDA content, a bioindicator of membrane damage caused by oxidative stress due to TEs, was increased in the leaves of the multicontamination group compared with the control group (by 23%). In contrast, in the roots, the MDA content decreased in the multicontamination group compared with the control group (by 22%). The results for the MDA content indicated the higher sensitivity of lettuce leaves to multicontamination toxicity compared with the roots. Furthermore, the 5-mC content, which was related to the level of global DNA methylation and possible epigenetic changes, showed the same trend in the leaves and roots of lettuce. The accumulation of As, Cd, Pb, and Zn in the multicontamination group led to an increase in the 5-mC content (Figure 2b). Compared with the control, the 5-mC content in the leaves and roots of the multicontamination group was 8.8 and 8.5-fold higher, respectively. An increase in DNA methylation in relation to the decrease in biomass indicated significant toxicity caused by multicontamination.

### 2.4. Free Amino Acid Content in Lettuce

**Figure 3:**
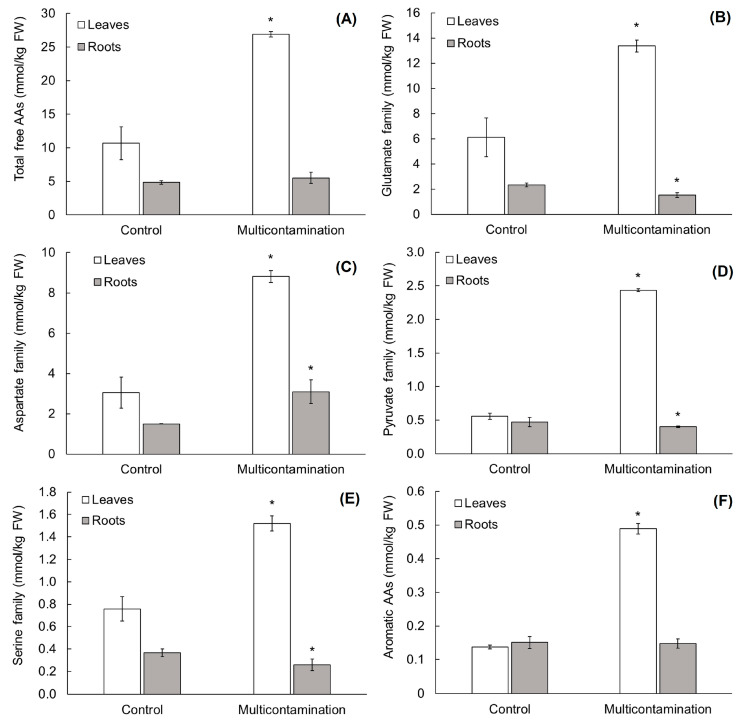
Change in the total content of free amino acids (**A**) and individual free amino acid families (**B**–**F**) in lettuce roots and leaves from the control and multicontaminated soil. Values represent means ± SD of four biological replicates. Asterisks indicate significant differences (*p* ≤ 0.05) between treatments (control × multicontamination) for lettuce organs based on Fisher’s LSD test.

In the lettuce roots and leaves, the change in free amino acid (AA) metabolism was evaluated in relation to the impact of As, Cd, Pb, and Zn contamination on the disturbance of N and C metabolism. All free AAs above the detection limit (20 free AAs and amides) were presented as the total content of free AAs (Figure 3A). Individual free AAs, except for free histidine, were divided according to the biosynthesis pathway of the following five families [16]: (i) glutamate family (Figure 3B), (ii) aspartate family (Figure 3C), (iii) pyruvate family (Figure 3D), (iv) serine family (Figure 3E), and (v) aromatic free AAs family (Figure 3F). Due to the separate biosynthetic pathway that was integrated with several other metabolic pathways, including tryptophan, free histidine was evaluated individually (Table S1).

In lettuce leaves, the content of free AA families showed the same trend as the total content of free AAs, with an increase in multicontamination (Figure 3A–F). Compared with the control, the total free AA content in the multicontamination group was 2.5-fold higher. Among the free AA families in the leaves of the control group and the multicontamination group, the highest abundance was determined for the glutamate family (Figure 3B), which included glutamate, glutamine, proline, ornithine, and γ-aminobutyric acid. The content of the glutamate family represented 57 and 50% of the total free AA content in the control and multicontamination groups, respectively. Multicontamination increased the glutamate family by 2.2 times compared with the control. The second family with the highest abundance was the aspartate family (Figure 3C), which included aspartate, asparagine, isoleucine, threonine, methionine, and lysine. The content of the aspartate family represented 28.5 and 33% of the total free AA content in the control and multicontamination groups, respectively. Compared with the control group, the multicontamination group increased the aspartate family 2.9-fold. The abundance of other families in the lettuce leaves varied by treatment and followed this order: (i) control/serine family (glycine and serine) > pyruvate family (alanine, leucine, and valine) > aromatic free AAs (phenylalanine, tyrosine, and tryptophane), and (ii) multicontamination/pyruvate family > serine family > aromatic free AAs. Multicontamination increased the content of serine, pyruvate, and aromatic AAs family by 2.0, 4.4, and 3.6 times, respectively (Figure 3C–F).

In lettuce roots, the total content of free AAs and free AA families showed a variable trend in response to multicontamination (Figure 3A–F). Compared with the control, the total content of free AAs was not significantly affected by multicontamination (Figure 3A). Similarly, a 2% reduction in the aromatic AAs family due to multicontamination was not statistically significant (Figure 3F), while the effect of multicontamination was statistically significant for other families (Figure 3B–E). The aromatic AA family in the roots of the control and multicontamination groups represented the group with the lowest abundance, i.e., 3% of the total free AA content. The abundance of other families varied depending on the treatment. In the control group, the highest abundance was observed for the glutamate family (48% of the total free AAs content) and decreased in the following order: aspartate family > pyruvate family > serine family. A different order was observed for the multicontamination group, with the highest abundance in the aspartate family (56% of the total free AAs content), followed by the glutamate, pyruvate, and serine families (Figure 3B–E). Furthermore, in the roots of the multicontamination group, the serine, pyruvate, and glutamate families decreased by 30, 15, and 34%, respectively, compared with the control group. In the case of the aspartate family in the roots of the multicontamination group, the opposite effect was observed, with a 107% increase compared with the control group (Figure 3C).

Compared with the roots, lettuce leaves accumulated 2.2 and 4.9 times higher levels of the total free AA content in the control and multicontamination groups, respectively (Figure 3A). Additionally, in the plant organs of both treatments, the aromatic AA family, which plays a major role in the regulation of plant development and defense responses, was the family with the lowest abundance. However, each free AA of this family—phenylalanine, tyrosine, and tryptophan—was increased by multicontamination in lettuce leaves (Table S1). Compared with the control group, phenylalanine, tyrosine, and tryptophan increased by 120, 173, and 507%, respectively, in the leaves of the multicontamination group, while the content of these free AAs reached similar levels in the lettuce roots. Together, these results indicated a disturbance of N and C metabolism caused by multicontamination and suggested the higher sensitivity of lettuce leaves to As, Cd, Pb, and Zn accumulation. Additionally, a change in free histidine content indicated the toxicity of multicontamination. This proteinogenic amino acid reached similar content in the lettuce leaves and roots of the control group (Table S1), while the content in the lettuce leaves and roots of the multicontamination group was 1.3- and 3.8-fold higher, respectively. Another free AA that is important for plant development and stress tolerance—lysine—was also increased by multicontamination in lettuce roots and leaves compared with the control (by 30 and 232%, respectively; Table S1).

### 2.5. Photosynthetic Parameters and Pigments of Lettuce

To evaluate the physiological response of lettuce leaves to As, Cd, Pb, and Zn contamination, the gas exchange parameters, water potential (WP), chlorophyll fluorescence (F_v_/F_m_), and the content of photosynthetic pigments were determined (Table 2). Among the gas exchange parameters, a significant change was observed in the transpiration rate (E) and stomatal conductance (g_s_), which were increased by As, Cd, Pb, and Zn accumulation. Compared with the control group, E and g_s_ of the multicontamination group increased by 76 and 90%, respectively. A similar effect was observed for the net photosynthetic rate (P_n_) and intercellular CO_2_ concentration (C_i_), but the change was not statistically significant (Table 2). In relation to these changes, the water use efficiency (WUE), determined as the ratio between P_n_ and E, decreased by 38% in the multicontamination group compared with the control group. This suggested that the accumulation of As, Cd, Pb, and Zn disturbed the water use of lettuce. Additionally, the results of WP (40% difference between treatments) indicated the significant influence of multicontamination on the water regime of lettuce (Table 2), and the WP value of multicontamination (−1.95 MPa) corresponded to medium water stress.

Additionally, F_v_/F_m_, which is commonly used to assess the physiological condition of plants, was reduced by 4% in lettuce leaves of the multicontamination group compared with the control group (Table 2). This result, together with the change in photosynthetic pigments, indicated the disturbance of photosynthesis caused by As, Cd, Pb, and Zn accumulation. As shown in Table 2, all photosynthetic pigments were reduced in the leaves of the multicontamination group. Compared with the control group, chlorophyll *a* (Chl *a*) content, chlorophyll *b* (Chl *b*) content, the ratio of chlorophyll *a* and *b* (Chl *a*/Chl *b*), the sum of chlorophyll *a* and *b* (Chl_total_), and the carotenoid content (Crt) decreased by 28, 21, 10, 26, and 14%, respectively.

### 2.6. Relationship between Toxic Elements and Physiological and Metabolic Parameters in Lettuce

Principal component analysis (PCA) was used to visualize the relationships between the content of toxic elements (As, Cd, Pb, and Zn) in the leaves and roots of lettuce and the observed physiological and metabolic parameters.

**Figure 4:**
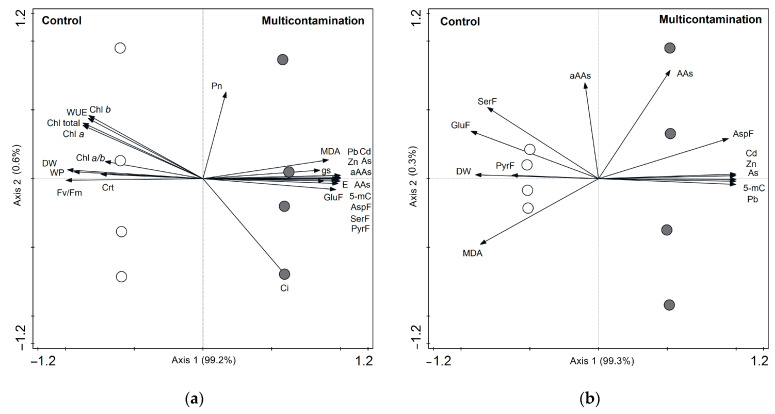
(**a**) Biplot of principal component analysis (PCA) for toxic element content and physiological and metabolic parameters in lettuce leaves; (**b**) biplot of PCA for toxic element content and physiological and metabolic parameters in lettuce roots. Abbreviations: 5-mC—5-methylcytosine; As, Cd, Pb, Zn—content of individual toxic elements in the biomass; aAAs—aromatic amino acids family; AAs—total content of free amino acids; AspF—aspartate family; Crt—carotenoids; C_i_—intercellular CO_2_ concentration; Chl *a*—chlorophyll *a*; Chl *b*—chlorophyll *b*; Chl *a*/*b*—ratio of chlorophyll *a* and *b*; Chl total—sum of chlorophyll *a* and *b*; DW—dry biomass; E—transpiration rate; F_v_/F_m_—maximum quantum yield of photosystem II; GluF—glutamate family; g_s_—stomatal conductance; MDA—malondialdehyde; P_n_—net photosynthetic rate; PyrF—pyruvate family; SerF—serine family; WUE—water use efficiency; WP—water potential. White circle—Control; grey circle—Multicontamination.

In the leaves, the PCA diagram (Figure 4a) showed that the first ordination axis explained 99.2% of all analyzed data variability. The symbols of the control group are located on the left side of the diagram and of the multicontamination group are on the right side. This finding indicated a significant impact of TEs contamination on the studied parameters. The angles of DW, photosynthetic pigments, WP, F_v_/F_m_, and WUE vectors indicated a negative correlation with the vector of the TEs content. On the other hand, the angles of MDA, 5-mC, C_i_, g_s_, E, free AAs, and AA families indicated a positive correlation with the vector of the TE content. The results of the correlation of parameters with the first and second ordination axes are presented in Table S2.

In the roots, the PCA diagram (Figure 4b) showed that the first ordination axis explained 99.3% of all analyzed data variability. Similarly, symbols are divided into opposite sides on the diagram, with the control group on the left side and the multicontamination group on the right side. The angles of DW, MDA, pyruvate, glutamate, and serine family vectors indicated a negative correlation with the vector of the TE content. On the other hand, the angles of free AAs, aspartate family, and 5-mC vectors indicated a positive correlation with the vector of the TE content. The results of the correlation of parameters with the first and second ordination axes are presented in Table S2.

## 3. Discussion

Many studies are available on the effect of single TE contamination on plant physiology and metabolism. However, most contaminated soils contain elevated concentrations of several TEs, and the negative effects of their combination can vary [17]. Among the TEs present in the experimental soil in our study, only Zn was considered essential for plant growth and metabolism. This element serves as a cofactor and enzyme activator [13,18]; however, an elevated content (up to 100 mg/kg) can lead to toxicity in plants [19]. Other elements in experimental soil—As, Cd, and Pb—are considered non-essential for plant metabolism and can cause severe toxic effects [13]. In relation to the content of these elements in the experimental soil, all elements in the multicontamination group were considered toxic. Therefore, the physiological and metabolic responses of lettuce to a combination of these TEs were observed in this study.

### 3.1. Impact of Toxic Element Multicontamination on Growth and Morpho-Anatomical Changes in Lettuce

TEs can accumulate in plants when they are grown in contaminated soils and negatively affect various aspects of plant life [18]. Various researchers have confirmed that leafy vegetables can accumulate a high content of different TEs, such as As, Cd, Pb, and Zn, in leaves [14,20,21,22,23,24,25]. There is limited information about the accumulation of TEs in the roots of leafy vegetables because leaves are the main edible parts for humans and, therefore, are the main focus of research. In our study, lettuce exhibited a high content of As, Cd, Pb, and Zn in the leaves of the multicontamination group; however, a higher content of TEs, especially Cd and Pb, was determined in the roots. Similarly, a higher accumulation of Cd [26] and Pb [27,28] was observed in lettuce roots. Additionally, the content of Cd and Pb in the lettuce leaves exceeded the permissible limits for leafy vegetables set by Regulation (EU) 2023/915 [29]. The accumulation of As, Cd, Pb, and Zn was evaluated using the BCF to assess the impact of elevated contents of other TEs in the soil on the accumulation of individual TEs in the lettuce. Particularly noteworthy was the case of Cd, where the BCF was >1 in the lettuce of the control, indicating efficient Cd transport from the soil to the lettuce biomass [26,30,31]. On the other hand, the BCF of Cd in the lettuce of the multicontamination group was <1, indicating the influence of interactions with other TEs in the soil and their bioavailability. The bioavailability of Cd in the soil was higher compared with Pb and other TEs [32]. Furthermore, the interaction between Cd and Zn significantly affected bioavailability of Cd for plants [33].

Plants take up TEs from their roots and can transfer TEs to different parts [19]. In our study, the lettuce of the multicontamination group translocated As and Zn from the roots to the leaves and showed values of TF, which reached values close to 1. Similarly, the TF of Cd from the control reached a value close to 1. In the case of Cd and Pb, TF values were lower than 1. As expressed by Sanjosé et al. [34], TF values lower than 1 or close to 1 indicate a trend to reduce TE content from roots to stems or leaves, a common behavior as, during their transportation through the plant, TEs become bound to the cell walls. Additionally, some TEs, such as Pb, are retained at the root level [34,35].

The accumulation of TEs is a serious threat to plant growth and development, leading to morphological and anatomical changes in the leaves and roots of plants (reviewed in [13,19]). These changes are related to oxidative stress caused by an imbalance of ROS due to the presence of TEs [36,37,38]. The harmful effects of multicontamination were observed in the growth of lettuce roots and leaves, resulting in a decrease in dry biomass, low TI, and visible symptoms of toxicity, such as leaf chlorosis and root browning. Chlorosis, as a common consequence of TE accumulation in plants, can indicate stress-induced senescence [13]. Compared with genetically programmed senescence (induction by internal age-dependent factors), stress-induced senescence is characterized by a faster process [39], during which the cell, tissue, or whole organ dies by degrees [40].

Lettuce roots, as the first organ that encounters TEs, were impacted by As, Cd, Pb, and Zn contamination. The influence of multicontamination was reflected by changes in the morphology and anatomy of roots, such as deformation, color change in tissues, xylem differentiation, and endodermis development. Similarly, a negative effect on the dry weight of lettuce and root growth rate [27,41], as well as a change in the branching pattern of roots, was observed for Pb [27]. A negative effect on the biomass growth of lettuce leaves and roots was also observed for Cd [26]. Akhter et al. [30] suggested that Casparian strips of lettuce were more permeable to Cd because cross-section analysis revealed no Cd accumulation at the endodermis in lettuce roots. Additionally, in lettuce roots, Cd caused a reduction in elongation [42].

### 3.2. Impact of Toxic Element Multicontamination on the Metabolic Response of Lettuce

As mentioned above, the accumulation of TEs in plants leads to oxidative stress through direct or indirect ROS production [36,37]. An increase in ROS can cause oxidative damage to the cell membrane system (lipid peroxidation), impairing the production of biomolecules, such as lipids, proteins, and nucleic acids, and even impairing plant photosynthesis and respiration, causing the death of plant cells in severe cases [43]. In relation to lipid peroxidation, MDA (one of the final products of polyunsaturated fatty acid peroxidation in cells) is widely used as a marker for determining the degree of injury to a stressed plant [44]. Typically, the more damaged the plant, the higher its MDA content, as indicated in studies focusing on plant responses to various stressors [45,46]. However, according to Morales and Munné-Bosch [44], MDA increases may represent acclimation processes rather than damage. This is because MDA can play a positive role by activating regulatory genes that are involved in plant defense and development, providing cell protection under oxidative stress conditions. These authors suggested that MDA may act as a protective mechanism rather than being an indicator of damage. In our study, the effect of TEs on the MDA content in lettuce varied depending on the plant organ. In lettuce leaves, an increase in MDA content indicated that higher As, Cd, Pb, and Zn accumulation in the multicontamination group impaired biomolecule production and plasma membrane permeability. The opposite effect of As, Cd, Pb, and Zn accumulation—a decrease in MDA content—was confirmed in lettuce roots, as also observed in response to various stressors in plants [46,47,48,49,50]. Gao et al. [51] suggested that a decrease in MDA was probably due to an increase in antioxidant enzyme activity, which helped reduce membrane damage. As oxidative stress increased, the MDA content accumulated, unless the plant induced active defense mechanisms, leading to a decrease in MDA content. The lower level of MDA in the roots of the multicontamination group was likely due to the detoxification of aldehydes through oxidation. The detoxification of these aldehydes through oxidation was attributed to aldehyde dehydrogenases. Aldehyde dehydrogenases expression was induced to control the levels of aldehyde compounds (such as MDA) by oxidizing them to their corresponding carboxylic acids. This process helped reestablish low cellular levels so that these compounds can serve as signals rather than cause harm to the cell [52]. In the roots, the results of MDA along with the observed morphological and anatomical changes, showed that lettuce was able to cope with the toxicity of multicontamination for a limited time due to its antioxidant activity.

Additionally, oxidative damage to biomolecules by ROS can lead to DNA damage and changes in DNA methylation in plants [36,40,53]. The latter is an important mechanism for epigenetic regulation of plant senescence and ageing since DNA methylation is an important regulator of biological processes, including growth, development, and protection from different plant stressors [36,40,54,55].

Generally, DNA methylation refers to the transfer of a methyl group onto the cytosine at position 5 to form 5-mC, which is used to indicate the level of global DNA methylation and potential epigenetic changes [55,56,57]. Stress-induced DNA methylation modifications and changes in 5-mC exhibit tissue, developmental, and stress specificity [53]. Most changes in DNA methylation induced by abiotic stress are transient and return to initial levels upon stress elimination [56]. In plants, the impact of TEs on DNA methylation is variable and depends on the plant species, type of TE, and its dose and treatment duration [55,57]. The results of our study revealed an increase in 5-mC in lettuce leaves and roots due to As, Cd, Pb, and Zn accumulation. Hypermethylation has been considered as one of the defense strategies against possible damage by TE products, allowing plants to survive in extreme environments [56]. Therefore, our results confirmed that the change in DNA methylation (characterized by 5-mC content), which was triggered by As, Cd, Pb, and Zn accumulation, can be considered one of the defense strategies in the lettuce from the multicontamination treatment. Consistent with our results, TEs caused an increase in DNA methylation, including 5-mC levels, in various plant species [58,59,60].

Another aspect of plant metabolism, which can be affected by the toxicity of TEs via ROS production, is N metabolism. According to Zhu et al. [61], N metabolism is central to the plant response to TEs, and its influence by TEs is related to the changes in free AA metabolism. Even though changes in AA metabolism in plants can vary widely due to the influence of TEs and the plant itself, several researchers have confirmed that AA increases in plant leaves and roots are caused by As, Cd, Pb, and Zn [3,61,62,63,64,65,66,67,68]. Consistent with these results, the TEs of the multicontamination group induced an increase in total free AA content in lettuce leaves, while the content in the lettuce roots was not significantly affected. In lettuce leaves, all AA families—glutamate, aspartate, pyruvate, serine, and aromatic AA family—copied the same trend as the total content of AAs, showing an increase due to multicontamination. However, the influence of multicontamination toxicity was variable in the roots, which matched the finding of Hildebrandt et al. [69], who stated that the changes in free AAs were dynamic and substantial in response environmental factors or developmental stages. Hildebrandt et al. [69] also stated that pool sizes of free AAs, which are smaller and more highly diverse compared with protein-bound AAs, depend not only on the ratio of AA biosynthesis and degradation but also on the biosynthesis and breakdown of proteins.

Additionally, changes in AA metabolism have been reported as one of the strategies for plant acclimation in coping with TE stress [19,70,71]. The effects of TEs on AA metabolism vary, and the extent of changes is influenced by the TE treatment, plant species, genotype, and part of the plant [61,62]. Our results indicated that the upregulation of free AAs in lettuce leaves was a response that helped lettuce cope with the toxicity of multicontamination and stress-induced senescence. Regarding the individual AAs, regulating the metabolism of certain AAs affected the level of other AAs due to the biosynthesis and catabolism of AAs acting as a substrate or intermediate and the strong relationship between the metabolic pathways of different AA families [72]. Among the free AA families of lettuce, the glutamate family was the most abundant, followed by the aspartate family. The abundance of these families was affected by multicontamination and changed in relation to the plant organ. The glutamate and aspartate family are strongly regulated under stress, including TE stress [66,72,73,74], and are connected to the primary energy metabolism of plants [75] and to cellular energy metabolism [76].

A similar phenomenon, with a different influence of treatment and plant organs, was observed for the remaining AA families in lettuce. Among these families, the pyruvate family, which is linked to primary C metabolism [77], was increased by multicontamination in lettuce leaves, while a decreasing trend was observed in lettuce roots. The results of the serine family, which is linked to the plant defense system under stress [78], copied the trend of the pyruvate family in lettuce leaves and roots. Despite that, the lowest abundance in lettuce was shown for the aromatic AA family. A high increase in all free AAs of this family by multicontamination indicated their involvement in the plant response to TE stress [16,61,79]. AA families, together with the change in free histidine and lysine, which are important free AAs involved in the plant stress response and growth and development [13,72,80,81], showed higher sensitivity to the toxicity of multicontamination in lettuce leaves compared with the roots via the significant disturbance of N and C metabolism. For plant adaptation to N and C status, and plant development and defense, regulation of the AA content, flux, and transport through the plant are critical [69]. Regarding the lettuce roots, a decrease in glutamate and pyruvate families indicated the use of AAs from these families in the detoxification of TEs, i.e., for phytochelatins synthesis [82]. Our results suggested that the regulation of free AAs in leaves of the multicontamination group was mainly linked to maintaining the N and C status during stress-induced senescence, while the regulation of free AAs in the roots was mainly linked to the detoxification of TEs due to the lettuce roots being the main storage of accumulated TEs.

### 3.3. Impact of Toxic Element Multicontamination on Photosynthesis and Water Potential of Lettuce

The toxicity of TEs affects the physiology of plants in many ways [13], e.g., TEs might cause the inhibition of assimilation, changes in gas exchange, respiration, or water balance. These changes are related to excessive ROS accumulation in plants, as they have been considered key signaling actors during senescence [37]. The induction of stress-induced senescence in plants can be indicated by changes in photosynthesis, fluorescence parameters, chlorophyll content, water content, and other parameters, such as growth parameters and lipid peroxidation [27]. A decrease in net CO_2_ assimilation was observed in lettuce due to Pb [41]. In contrast to the effect of Pb alone, our results indicated the difference between single and combined contamination. The toxicity of multicontamination affected the gas exchange parameters and other parameters related to the photosynthesis of lettuce. A significant change was observed for E, g_s_, and C_i_, which increased under multicontamination stress. These results are linked to the increase in the pyruvate family in lettuce leaves due to the relationship of this family to plant respiration under stress [83]. According to Avezedo Neto et al. [83], respiration increases to sustain the higher energy demand of stress conditions or to provide C skeletons for the photorespiratory cycle. In addition, the serine family is linked to the photorespiratory pathway and can indicate alternations in C flow [84]. Both families were increased by As, Cd, Pb, and Zn accumulation in leaves but decreased in roots, indicating a higher demand for N and C in lettuce leaves.

The lower TE content in leaves compared with roots, indicated that the impact of multicontamination toxicity on leaves was also associated with root damage and disruption in water uptake. A change in root anatomy, especially the disturbed function of Casparian strips, leads to a reduction in water and nutrient uptake [85,86]. Furthermore, the disruption in the water regime of lettuce was indicated by the results of WP, which corresponded to medium water stress. Water plays an irreplaceable role in the plant assimilation process. The lack of water can lead to limited light and dark parts of photosynthesis and reduced chlorophyll content. A decrease in the content of photosynthetic pigments leads to reduced photosynthetic activity and altered fluorescence [87,88], and a continuous decrease in chlorophylls indicates degradation of the photosynthetic apparatus [27,89].

Our results confirmed the harmful effect of multicontamination on the F_v_/F_m_ of lettuce. A decrease in the F_v_/F_m_ value indicated that part of the reaction center of the PSII photosystem was damaged or deactivated [90], likely due to accelerated leaf senescence [91]. The reduced activity of the reaction center resulted in excess light energy, which could be converted into heat [92,93]. Plants respond to this condition by increasing transpiration and, in connection with this, by increasing stomatal conductance [94]. A similar response was observed for multicontamination in lettuce, as indicated by the decreased value of WUE, which suggested the inefficiency of the water regime.

Additionally, our results confirmed the negative effect of multicontamination on photosynthetic pigments, which was shown as a decrease in chlorophylls and carotenoids. Our results matched the findings reported in several studies [60,95,96,97] regarding reduction in photosynthetic pigment in the leaves of plants exposed to TEs. Regarding the carotenoids, these pigments play a protective role against ROS; however, a common response to TEs toxicity is a decrease in carotenoids, which increases the sensitivity of photosynthesis to adversity [95]. In our study, the reduced carotenoid content in the multicontamination lettuce was associated with this response. Plant response to TEs stress is regulated by a set of hormones and signaling molecules, which includes metabolites originating from carotenoids. The initial step in the formation of these carotenoid-derived signals is an oxidation that yields a carbonyl group containing molecules called apocarotenoids. Further modifications of the primary cleavage products lead to the formation of hormones, mainly abscisic acid [98]. Abscisic acid coordinates plant response to abiotic stress. It regulates the water uptake by plants by controlling the closure of stomata, which mediate the uptake and release of oxygen and carbon dioxide for respiration and photosynthesis [99].

## 4. Materials and Methods

### 4.1. Plants and Soil

The pot experiment was carried out in a greenhouse (natural light conditions; temperature of 20–23 °C during the day and 15–18 °C at night; relative humidity ~60%) using a randomized design with four replications for each treatment. The soil used in the experiment was collected (0–20 cm) from two localities in the Czech Republic (Table 3). Two treatments were defined based on the determined TE contents, which were compared with Czech legislation limits for the pseudo-total content in light-textured and other soils: 15 and 20 mg/kg for As, 0.4 and 0.5 mg/k for Cd, 55 and 60 mg/kg for Pb, and 105 and 120 mg/kg for Zn [100]. Soil from the locality Suchdol was used as non-contaminated soil (the control group) and soil from the locality Litavka with an elevated content of As, Cd, Pb, and Zn was used as the multicontaminated soil (the multicontamination group). Each pot (3 L) was filled with 2.5 kg of soil and mixed with nutrients in doses of 0.5 g N, 0.16 g P, and 0.4 g K per pot (applied as NH_4_NO_3_ and K_2_HPO_4_ solutions) for the relevant treatments.

Lettuce (*Lactuca sativa* var. *capitata* L.) was used as the model plant and purchased as pre-cultivated plants (four true-leaf stage) from the garden center of the Czech University of Life Sciences Prague (Prague, Czech Republic). Plants were placed in the pots with soil (1 plant per pot) and regularly irrigated. Lettuce was harvested after 45 days of growth in pots and divided into roots and leaves. Each organ was washed with distilled water, blotted dry with filter paper, and weighed. The samples were partitioned for further analysis. The corresponding amount of fresh biomass (in the case of leaves, material without chlorosis and necrosis) for the analysis of metabolites (MDA, free AAs), and DNA isolation was immediately frozen in liquid nitrogen and stored at −80 °C. The remaining amount of material was weighted, oven-dried at 40 °C to a constant weight, used for determination of dry biomass, and homogenized for element analysis.

### 4.2. Microscopic Observation

To observe the structural symptoms of combined TE toxicity, cross-sections through the tap and lateral roots of lettuce were performed (without staining and at 100× magnification) using a Nikon Eclipse 50i microscope with a Nikon DS-Fi2 camera (Nikon Corporation, Tokyo, Japan).

### 4.3. Toxic Element Determination

The content of As, Cd, Pb, and Zn in the dry biomass of lettuce (0.5 ± 0.05 g) was determined by an Agilent 720 inductively coupled plasma optical emission spectrometer (ICP-OES; Agilent Technologies Inc., Santa Clara, CA, USA) after low-pressure microwave digestion [3]. Homogenized material was digested in 10 mL of a mixture of HNO_3_ and H_2_O_2_ (4:1, *v*/*v*) at 120–180 °C in an Ethos 1 device (MLS GmbH, Leutkirch im Allgäu, Germany). After cooling, the digested sample was diluted to 50 mL with demineralized water. The limit of detection of As, Cd, Pb, and Zn was 0.03, 0.001, 0.02, and 0.002 mg/L, respectively. The limit of quantification of As, Cd, Pb, and Zn was 3, 0.1, 2, and 0. 2 mg/kg, respectively. Certified reference materials (CRM NIST 1573a tomato leaves and CRM NIST 1570a spinach leaves, Analytika^®^, Prague, Czech Republic) were mineralized under the same conditions for quality assurance.

### 4.4. Factors Calculation

The plant dry biomass was evaluated by the tolerant index (TI) according to Antoniadis et al. [4]. The TI was calculated for determination of plant yield decrease as a result of multicontamination by the equation:

TI = DB_Multi_/DB_Cont_, (1)

where DB_Multi_ is dry biomass of the multicontamination group and DB_Cont_ is dry biomass of the control group. Generally, values of TI approaching zero indicate plant sensitivity to contamination, while TI equals unity when plants are unaffected by and thus tolerate contamination.

The content of the individual TEs in leaves and roots of lettuce was evaluated by the bioconcentration factor (BCF) and translocation factor (TF) according to Sanjosé et al. [34]. The BCF and TF were calculated for determination of accumulation and translocation of the individual TEs in lettuce by the equation:

BCF = TE_organ_/TE_soil_, (2)

TF = TE_leaves_/TE_roots_, (3)

where TE_organ_ is the content of individual TE in lettuce leaves (TE_leaves_) or roots (TE_roots_) and TE_soil_ is the content of individual TE in the soil. Values of BCF greater than 1 indicate a higher content of TEs in plant biomass than the available content in the soil and vice versa. Also, values of TF greater than 1 indicate a high translocation of TEs from roots to the other organs of plant [4,34].

### 4.5. Malondialdehyde and 5-Methylcytosine Determination

The MDA content in the fresh biomass of lettuce (0.4 ± 0.05 g) was determined based on a modified thiobarbituric acid (TBA) determination method [60]. Lettuce material was homogenized with liquid nitrogen and 80% ethanol and centrifuged in 2 mL microcentrifuge tubes for 5 min at 6000 rpm. Aliquots of 0.7 mL of each supernatant were mixed with 0.7 mL of 0.65% TBA in 20% trichloroacetic acid (TCA) and 0.01% butylated hydroxytoluene (BHT), and a second set of 0.7 mL samples was mixed with 0.7 mL of 20% TCA and 0.01% BHT. The microcentrifuge tubes were incubated at 95 °C for 25 min and, after cooling, centrifuged for 5 min at 6000 rpm. The absorbance was read at 440, 532, and 600 nm on a UV–VIS spectrophotometer (Evolution 201, Thermo Scientific Inc., Waltham, MA, USA).

The percentage of 5-methylcytosine (relative DNA methylation status) was determined in the fresh lettuce biomass. Plant DNA was isolated using a NucleoSpin Plant II Molecular Kit (Macherey–Nagel GmbH & Co. KG, Düren, Germany), as described by Zemanová et al. [66]. The global DNA methylation status of DNA was determined from 100 ng of isolated DNA using a MethylFlash Methylated DNA Quantification Kit (Fluorometric; Epigentek Group Inc., Farmingdale, NY, USA) according to the manufacturer’s instructions. A spectrophotometer (Tecan Infinity M200, Tecan Deutschland GmbH, Salzburg, Austria) with excitation at 530 nm was used to measure the fluorescence at 590 nm.

### 4.6. Free Amino Acid Determination

The free AA content in the fresh material of lettuce roots and leaves (0.5 ± 0.05 g) was determined using a Hewlett Packard 6890N/5975 MSD gas chromatography–mass spectrometry system (GC-MS; Agilent Technologies Inc., Santa Clara, CA, USA) with a ZB-AAA 10 m × 0.25 mm AA analysis GC column after derivatization of extracts by an EZ:faast kit (Phenomenex, Torrance, CA, USA). Extraction was performed as previously described [3].

### 4.7. Photosynthetic Parameters and Pigment Determination

Photosynthetic parameters—net photosynthetic rate (P_n_), transpiration rate (E), stomatal conductance (g_s_), and intercellular CO_2_ concentration (C_i_)—were determined using the portable gas exchange system LCpro+ (ADC BioScientific, Ltd., Hoddesdon, UK). All measurements were conducted between 8:00 and 13:00 h. The duration of each measurement was 10 min after the establishment of steady-state conditions inside the measurement chamber. The conditions in the chamber were as follows: 25 °C, ambient CO_2_ concentration of 550 ± 50 μL/L, air flow rate of 205 ± 30 μmol/s, and irradiance of 650 ± 50 μmol(photon)/m^2^/s of photosynthetically active radiation. The fully expanded young leaves (leaves of medium age from the middle of the head of lettuce) were selected for measurement. From the measured data, the WUE was calculated as WUE = P_n_/E.

For chlorophyll fluorescence, the maximum quantum yield of PSII (F_v_/F_m_) was calculated as F_v_/F_m_ = (F_m_ − F_0_)/F_m_. The parameters (F_m_, F_0_) were measured using a portable fluorometer (OS1-FL; Opti-Sciences, ADC, BioScientific, Ltd., Hoddesdon, UK). A fresh leaf was shaded for 20 min using clips to set up a dark-adapted state and then irradiated using a 660 nm solid-state light source, with filters blocking radiation longer than 690 nm. The saturation of the measured photosystem was achieved using a filtered 35 W halogen lamp (350–690 nm) with a pulse of 15,000 μmol/m^2^/s for 0.8 s.

The leaf water potential (WP) was measured psychometrically using a dew point PotentiaMeter (Decagon Devices, Inc., Pullman, WA, USA). The lettuce leaves were placed in disposable syringes. The air was drawn off the syringe, and the syringe was tightly closed with Parafilm. The specimen was frozen at −18 °C and then thawed, and the sap was pushed into the measuring chamber of the PotentiaMeter.

The pigment content—chlorophyll *a* (Chl *a*), chlorophyll *b* (Chl *b*), the sum of chlorophyll *a* and *b* (Chl_total_), and carotenoid (Crt) content—was analyzed spectrophotometrically from the extract prepared, as previously described [60]. Fresh leaf segments (0.5 cm^2^) were incubated in the dark (24 h) in 1 mL dimethylformamide, shaken, and measured using a UV–VIS spectrophotometer (Evolution 210, Thermo Fisher Scientific Inc., Waltham, MA, USA). The absorbance of the extract was measured at wavelengths of 480, 646.8, and 663.8 nm. Absorbance values at 710 nm were subtracted from these measurements. From these data, the pigment contents were calculated using equations from Porra et al. [101] for chlorophyll and from Wellburn [102] for Crt. The pigment content was normalized by the leaf area.

### 4.8. Statistical Analyses

Statistica 12.0 software (StatSoft, Tulsa, OK, USA) was used for the statistical processing of the results. The data from the technical replicates were averaged for four independent biological repeats (pots) of each treatment and expressed as mean values and standard deviation (SD). All data were checked for homogeneity of variance and normality (by Levene’s and Shapiro–Wilk tests). The data met assumptions for the use of a one-way analysis of variance (ANOVA). ANOVA with Fisher’s LSD test (*p* ≤ 0.05) was used to identify statistically significant differences among the treatments of individual plant organs. The relationships between the content of toxic elements and the observed physiological and metabolic parameters of individual plant organs were assessed by PCA using the CANOCO 5.1 program (Microcomputer Power, Ithaka, NY, USA). In the PCA diagram (biplot), the length and direction of the vectors indicated the strength of the vector effect and the correlation between vectors. A long vector for a particular variable indicated that it greatly affected the results of the analysis, while the opposite was true for a short vector. An angle of <90° between the vectors indicated that they were positively correlated. An angle of >90° between two vectors indicated that they were not positively correlated.

## 5. Conclusions

The harmful effects of TEs in multicontaminated soil were evident in the inhibition of lettuce growth, with visible symptoms of toxicity, such as leaf chlorosis and root browning, indicating stress-induced senescence and morphological and anatomical changes in the roots, such as deformation, color changes in tissues, xylem differentiation, and endodermis development. The markers for the degree of ROS stress and injury to a plant—MDA and DNA methylation—clearly confirmed oxidative stress in lettuce. In the lettuce leaves, the increase in the total content of free AAs was related to the influence of TEs on the disturbance of N and C metabolism. In the leaves, the results suggested a link between changes in the pyruvate and serine families and photorespiration. Other photosynthetic parameters of lettuce leaves reflected plant sensitivity to stress conditions, especially the results of photosynthetic pigments indicating the disturbance of photosynthesis and accelerated stress-induced senescence. Our results confirmed that the toxicity of TEs in multicontaminated soil significantly altered physiological and metabolic processes in plants by damaging their antioxidant systems.

## Figures and Tables

**Table 1:** Content of toxic elements (TEs, mg/kg DW) and bioconcentration factor (BCF, defined as the ratio of TE content in leaves or roots to TE content in the soil) in lettuce biomass from the control and multicontaminated soil. Values represent the mean ± SD of four biological replicates. Asterisks indicate significant differences (*p* ≤ 0.05) between treatments (control × multicontamination) for lettuce leaves and roots within the line based on Fisher’s LSD test. Number signs indicate significant differences (*p* ≤ 0.05) between organs (leaves × roots) of individual treatment within the column based on Fisher’s LSD test. DW—dry weight, ND—value was below limit of detection (As 3 mg/kg DW and Pb 2 mg/kg DW).

TEs	Control (mg/kg DW)	BCF	Multicontamination (mg/kg DW)	BCF
As—leaves	ND	-	7.95 ± 0.72	0.03
Cd—leaves	0.92 ± 0.04	2.30	19.08 ± 0.44 *	0.51
Pb—leaves	ND	-	18.16 ± 3.44	0.01
Zn—leaves	22.76 ± 0.73	0.27	669.37 ± 9.50 *	0.19
As—roots	ND	-	8.56 ± 0.05	0.03
Cd—roots	0.96 ± 0.09	2.39	27.38 ± 3.14 *#	0.73
Pb—roots	ND	-	28.60 ± 0.74 #	0.01
Zn—roots	56.63 ± 1.65 #	0.66	688.74 ± 55.07 *	0.20

**Table 2:** Water potential, gas exchange parameters, chlorophyll fluorescence, and photosynthetic pigments of lettuce from the control and multicontaminated soil. P_n_—net photosynthetic rate, E—transpiration rate, g_s_—stomatal conductance, C_i_—intercellular CO_2_ concentration, WUE—water use efficiency, F_v_/F_m_—maximum quantum yield of photosystem II, Chl *a*—chlorophyll *a*, Chl *b*—chlorophyll *b*, Chl *a*/Chl *b*—ratio of chlorophyll *a* and *b*, Chl_total_—sum of chlorophyll *a* and *b*, Crt—carotenoids, WP—water potential. Values represent means ± SD of four biological replicates. Asterisks indicate significant differences (*p* ≤ 0.05) between treatments (control × multicontamination) within the line based on Fisher’s LSD test.

Parameter	Control	Multicontamination
WP (MPa)	−1.39 ± 0.06	−1.95 ± 0.15 *
P_n_ (μmol CO_2_/m^2^/s)	8.20 ± 1.93	8.81 ± 2.03
E (mmol H_2_O/m^2^/s)	1.51 ± 0.18	2.66 ± 0.45 *
g_s_ (mol H_2_O/m^2^/s)	0.11 ± 0.03	0.21 ± 0.04 *
C_i_ (μmol CO_2_/mol)	266.29 ± 20.96	296.60 ± 25.92 *
WUE (μmol CO_2_/mmol H_2_O) ^1^	5.39 ± 0.83	3.35 ± 0.72 *
F_v_/F_m_ ^2^	0.78 ± 0.002	0.75 ± 0.002 *
Chl *a* (mg/m^2^)	156.66 ± 17.71	112.90 ± 10.16 *
Chl *b* (mg/m^2^)	47.59 ± 4.36	37.94 ± 3.28 *
Chl *a*/Chl *b*	3.32 ± 0.23	3.02 ± 0.08 *
Chl_total_ (mg/m^2^)	204.26 ± 21.27	150.84 ± 13.30 *
Crt (mg/m^2^)	36.83 ± 3.51	30.79 ± 2.75 *

^1^ WUE = P_n_/E. ^2^ F_v_/F_m_ = (F_m_ − F_0_)/F_m_.

**Table 3:** Basic characteristics and toxic element content of experimental soils.

Parameter	Control	Multicontamination
Locality	Suchdol (50°8′8″ N, 14°22′43″ E)	Litavka (49°43′ N, 14°0′ E)
Soil type	Haplic chernozem	Gleyic fluvisol
Soil texture	Silt loam	Sandy loam
pH_H2O_	7.1 ± 0.1	5.4 ± 0.1
CEC (mmol_(+)_/kg) ^1^	230.1 ± 5.0	109.0 ± 31.9
C_organic_ (%)	1.8 ± 0.3	3.6 ± 0.4
DOC (mg/kg) ^2^	153.0 ± 3.4	317.3 ± 19.3
As_pseudo-total_ (mg/kg)	18.1 ± 1.0	283.9 ± 7.7
Cd_pseudo-total_ (mg/kg)	0.4 ± 0.01	37.4 ± 1.1
Pb_pseudo-total_ (mg/kg)	32.1 ± 0.7	2361.2 ± 32.4
Zn_pseudo-total_ (mg/kg)	85.5 ± 2.5	3496.6 ± 60.2

^1^ CEC—cation exchange capacity [14,21,22,23,24,25]. ^2^ DOC—dissolved organic carbon.

## Data Availability

The data presented in this study are available in the article.

## Supplementary Materials

The following supporting information can be downloaded at: https://www.mdpi.com/article/10.3390/plants13101356/s1, Table S1: Content (mmol/kg FW) of selected free amino acids in lettuce leaves and roots from the control and multicontaminated soil. Table S2: Results of principal component analysis (PCA) of physiological and metabolic parameters in lettuce—correlation between vectors of parameters and axes of biplot.
