# Supplementary material for: Multicontamination Toxicity Evaluation in the Model Plant Lactuca sativa L
Source: Plants (Basel). 2024 May 14;13(10):1356. doi: 10.3390/plants13101356 (PMC11125215; doi:10.3390/plants13101356)
Supplement: Supplementary file 1 [file plants-13-01356-s001.zip › Table S1.pdf]

Table S1 Content (mmol/kg FW) of selected free amino acids in lettuce leaves and roots from both the control and multicontaminated soil. Values represent means ± SD. Asterisks indicate significant differences ($p \leq 0.05$) between treatments (Control × Multicontamination) for lettuce organs based on Fisher's LSD test.

**Parameter**	**Control**	**Multicontamination**
Phenylalanine – leaves	0.045 ± 0.003	0.100 ± 0.002*
Tryptophan – leaves	0.042 ± 0.002	0.255 ± 0.012*
Tyrosine – leaves	0.050 ± 0.002	0.134 ± 0.003*
Histidine – leaves	0.072 ± 0.002	0.272 ± 0.014*
Lysine – leaves	0.105 ± 0.007	0.346 ± 0.013*
Phenylalanine – roots	0.030 ± 0.004	0.030 ± 0.004
Tryptophan – roots	0.068 ± 0.010	0.077 ± 0.008
Tyrosine – roots	0.053 ± 0.003	0.041 ± 0.002*
Histidine – roots	0.061 ± 0.005	0.081 ± 0.010*
Lysine - roots	0.084 ± 0.002	0.108 ± 0.006*
