# Supplementary material for: Multicontamination Toxicity Evaluation in the Model Plant Lactuca sativa L
Source: Plants (Basel). 2024 May 14;13(10):1356. doi: 10.3390/plants13101356 (PMC11125215; doi:10.3390/plants13101356)
Supplement: Supplementary file 1 [file plants-13-01356-s001.zip › Table S2.pdf]

Table S2 Results of principal component analysis (PCA) of physiological and metabolic parameters in lettuce - correlation between vectors of parameters and axes of biplot. Abbreviations: 5-mC – 5-methylcytosine; As, Cd, Pb, Zn – content of individual toxic elements in the biomass; aAAs – aromatic amino acids family; AAs – total content of free amino acids; AspF – aspartate family; Crt – carotenoids; C_i – intercellular CO_2 concentration; Chl a – chlorophyll *a*; Chl b – chlorophyll *b*; Chl a/b – ratio of chlorophyll *a* and *b*; Chl total – sum of chlorophyll *a* and *b*; DW – dry biomass; E – transpiration rate; F_v/F_m – maximum quantum yield of photosystem II; GluF – glutamate family; g_s – stomatal conductance; MDA – malondialdehyde; P_n – net photosynthetic rate; PyrF – pyruvate family; SerF – serine family; WUE – water use efficiency; WP – water potential.

**Parameter**	**Leaves**		**Roots**	
	**Axis 1**	**Axis 2**	**Axis 1**	**Axis 2**
5-mC	0.9988	-0.0003	-0.8952	0.0276
As	0.9923	-0.0005	0.9982	-0.0192
Cd	0.9996	0.0230	0.9998	-0.0057
Pb	0.9995	0.0238	0.9983	0.0214
Zn	1.0000	0.0079	0.9976	-0.0419
aAAs	0.9985	-0.0178	0.5212	0.7887
AAs	0.9825	-0.0363	0.9994	0.0330
AspF	0.9839	0.0017	-0.9266	0.3449
Crt	-0.7425	0.0324	-	-
C_i	0.5970	-0.6993	-	-
Chl a	-0.8741	0.3883	-	-
Chl b	-0.8304	0.4593	-	-
Chl a/b	-0.7125	0.1250	-	-
Chl total	-0.8732	0.4051	-	-
DW	-0.9852	0.0639	-0.8600	-0.4782
E	0.8806	-0.0168	-	-
F_v/F_m	-0.9952	-0.0122	-	-
GluF	0.9660	-0.0797	-0.0991	0.6973
g_s	0.8497	0.0610	-	-
MDA	0.9128	0.1364	-0.6298	0.0246
P_n	0.1674	0.6300	-	-
PyrF	0.9990	0.0055	-0.8080	0.5179
SerF	0.9776	-0.0090	0.9437	0.2924
WUE	-0.8347	0.4384	-	-
WP	-0.9393	0.0500	-	-
Eigenvalue	0.9922	0.0054	0.9930	0.0035
Variability (%)	99.22	99.77	99.30	99.64
